# Complete Genome Sequence of a Bovine Viral Diarrhea Virus Subgenotype 1h Strain Isolated in Italy

**DOI:** 10.1128/genomeA.01697-16

**Published:** 2017-02-23

**Authors:** Moira Bazzucchi, Luigi Bertolotti, Monica Giammarioli, Cristina Casciari, Elisabetta Rossi, Sergio Rosati, Gian Mario De Mia

**Affiliations:** a Istituto Zooprofilattico Sperimentale dell’Umbria e delle Marche, Perugia, Italy; b Dipartimento di Scienze Veterinarie, Università degli Studi di Torino, Grugliasco, Turin, Italy; c Dipartimento di Medicina Veterinaria, Università degli Studi di Perugia, Perugia, Italy

## Abstract

We sequenced the complete genome of bovine viral diarrhea virus (BVDV) strain UM/126/07. It belongs to subgenotype 1h. The complete genome is composed of 12,196 nucleotides organized as one open reading frame encoding 3,898 amino acids. This is the first report of a full-length sequence of BVDV-1h.

## GENOME ANNOUNCEMENT

Bovine viral diarrhea virus (BVDV) is an important pathogen of ruminants (1, 2), causing severe economic losses to the cattle industry (3, 4). Based on antigenic and nucleotide differences, two genotypes (BVDV1 and BVDV2) have been described in the past. In addition, genetically distinct isolates may be members of a third putative species, BVDV3 (HoBi-like viruses) (5). Twenty-one subgenotypes (BVDV-1a to BVDV-1u) have been reported in the world (6–11). Italy is one of the countries with the highest genetic diversity of BVDV (8). Subgenotype 1h has been sporadically reported in the cattle population (8, 12). A high level of BVDV genetic heterogeneity is mainly attributable to the absence of any BVDV systematic control measure. Therefore, it is important to know which subtypes are circulating and how their prevalence is changing over time. In the current study, we determined the full-length genome sequence of a BVDV-1h strain.

The UM/126/07 isolate was collected in 2007 from a persistently infected animal. The viral RNA was extracted with the QIAamp viral RNA minikit after appropriate enzymatic digestion. The quality of the total RNA was verified using a 2200 TapeStation RNA Screen Tape device (Agilent Technologies, Santa Clara, CA, USA) and its concentration ascertained using an ND-1000 spectrophotometer (NanoDrop, Wilmington, DE). Libraries were prepared by PGP with the Illumina TruSeq RNA sample prep kit (Illumina, Inc.), according to the manufacturer’s protocol. The double-stranded cDNA (ds-cDNA) was end-repaired and adenylated; an Illumina adapter was added, as indicated in the TruSeq RNA protocol. The prepared libraries were evaluated with the high-sensitivity D1000 Screen Tape (Agilent Tape Station 2200). The indexed libraries were quantified with the ABI9700 quantitative PCR (qPCR) instrument using the Kapa library quantification kit in triplicate, according to the manufacturer’s protocol (Kapa Biosystems, Woburn, MA, USA). Five microliters of the pooled library at a final concentration of 2 nM was used for sequencing using Illumina MiSeq. The reads generated were analyzed using an assembly *de novo* approach and by using the analytical tools ABySS, Velvet, and Mira, integrated into Geneious 9.1.2. By means of this assembly procedure, the complete sequence of the isolate was obtained.

The complete genome of the strain UM/126/07 comprises 12,196 nucleotides (nt), with 5′ and 3′ untranslated regions (UTRs) of 348 nt and 152 nt, respectively. The single large open reading frame encodes 3,898 amino acids. Compared to other subgenotypes, the virus shares only 81.56% (subtype 1m) to 77.29% (subtype 1a) nucleotide similarity with other published full-length BVDV-1 genomes. The large open reading frame encodes four structural proteins (C [nt 852 to 1157], E^rns^ [nt 1196 to 1876], E1 [nt 1839 to 2423], and E2 [nt 2424 to 3545]) and seven nonstructural proteins (N^pro^ [nt 348 to 851], p7 [nt 3546 to 3755], NS2/3 [nt 3756 to 7163], NS4A [nt 7164 to 7355], NS4B [nt 7356 to 8396], NS5A [nt 8397 to 9884], and NS5B [nt 9884 to 12041]). The publication of the first full-length BVDV-1h sequence will improve knowledge of diagnostics and disease control, giving valuable molecular data to trace the source of infection, mode of transmission, and vaccine selection.

### Accession number(s).

The genomic sequence of BVDV UM/126/07 has been deposited in GenBank under the accession no. LT631725.
